# Effects of sporulation times in liquid or on plates on *Bacillus subtilis* spore resistance, germination, inner membrane fluidity and permeability, and core contents

**DOI:** 10.1128/jb.00389-25

**Published:** 2025-11-14

**Authors:** Faith Ye, Dhruv Suryadevara, William J. Bannon, Jane Setlow Anetsky, Zaara Khan, Nicole Eugenio, James Wicander, George Korza, Peter Setlow

**Affiliations:** 1 Department of Molecular Biology and Biophysics, UConn Health 705913, Farmington, Connecticut, USA; The Ohio State University, Columbus, Ohio, USA

**Keywords:** spore germination, spore resistance, spores, *Bacillus*, spore killing

## Abstract

This study aimed to determine reasons that longer sporulation times in liquid or on plates have large effects on *Bacillus subtilis* spore germination and resistance. *B. subtilis* spores were prepared for 3/30/60 days in liquid or on plates and their germination, resistance, levels of core water and Ca^2+^-dipicolinic acid (CaDPA), and inner membrane (IM) fluidity and permeability were measured. Liquid spores of 3/30/60 days had no differences in wet heat resistance, while plate spores of 30/60 days had higher wet heat resistance than 3-day plate spores. There were minimal increases in 3- to 60-day liquid spores’ resistance to UV radiation and chemicals, while plate spores of 30/60 days had increased resistance to chemicals. Germinant receptor (GR) germination with L-valine was identical with 3- to 60-day liquid or plate spores, while plate spores of 30/60 days germinated faster than 3-day spores with the L-asparagine, D-glucose, D-fructose, and KCl mixture. Three-day liquid or plate spores germinated faster than 30- and 60-day spores with the GR-independent germinant, dodecylamine, and 30-day plate spores had lower IM permeability and higher IM rigidity than 3-day plate spores or 30-day liquid spores. However, levels of two spore core components’ modulating spore wet heat resistance, water, and CaDPA were identical in spores of 3/30/60 days. Thus, increased IM rigidity and decreased IM permeability appear to be major factors in increased resistance of spores prepared on plates for long periods. However, precisely how these longer times cause increased IM rigidity and lower permeability, but not with spores prepared in liquid, is not clear.

**IMPORTANCE:**

Cells of some Bacillota cause food spoilage and human diseases. These organisms’ ability to do this is exacerbated by forming hard-to-kill dormant spores because of their resistance and ability to come “back to life” in germination. We examined two *Bacillus subtilis* sporulation parameters, liquid versus solid media and sporulation time, measuring effects on spore resistance and germination. We found (i) an effector of spore heat resistance, core water content, is not changed by different sporulation media or times; and (ii) spores’ IM becomes more rigid and less permeable in spores made on solid media and for longer times. This knowledge may influence how spores are prepared as probiotics or as standards for analyses of autoclave function.

## INTRODUCTION

Spores of Bacillota are dormant and resistant to many killing treatments (1, 2). While these spores can survive for years, they can return to life rapidly in spore germination and then outgrowth, which convert spores into growing cells (3). Since cells of some growing or stationary phase spore formers cause food spoilage, severe diseases, and intoxications (1), there is continued interest in how spore properties are acquired and affect spore resistance and germination. Many sporulation variables affect spore resistance and germination, including (2, 4–12) (i) temperature, (ii) water activity, (iii) ion contents in media, (iv) richness of media, and (v) liquid versus solid media. There have also been reports indicating that sporulation time affects spore properties, with increases in spore wet heat resistance with increasing sporulation times (7–9). For many of these sporulation variables, the mechanisms behind changes in spore properties have been identified (5, 9), but for others, they have not. The latter is especially the case for the effects of liquid versus solid sporulation media and for different times. Consequently, we examined in detail the effects of these two sporulation variables on multiple spore resistance properties and how well spores can germinate with different germinants. These analyses, as well as effects of sporulation time on spore inner membrane (IM) fluidity, permeability, and core water and CaDPA contents, all suggest and rule out specific explanations for changes in spore resistance and germination with different sporulation times in liquid or on plates. However, specific causes of some effects on spore properties are still not well understood.

## MATERIALS AND METHODS

### *B. subtilis* strain used and spore preparation and purification

The *B. subtilis* strain used in the work is PS832, a wild-type laboratory 168 strain. Its sporulation was at 37°C with 2× Schaeffers–glucose (2×SG) medium (13), either shaking in liquid or on agar plates. Sporulation plates of each species were all held in lightly sealed sleeves in incubators at appropriate temperatures. With sporulations in liquid (250 mL in 500 mL flasks capped with aluminum foil), the height of the 250 mL in flasks at time zero was marked with a line, and every 5 days, sterile 37°C water was added to reach the line. Spores were harvested from liquid medium and washed several times with ~30 mL of 4°C sterile water. The washed spores’ purification (14) was by multiple sonication treatments over 3–4 days with subsequent washing by centrifugation and finally by centrifugation through a solution of 50% Nycodenz in which spores’ pellet, while debris, germinated spores, and growing or sporulating cells float. The Nycodenz in pelleted spores was removed by washing with cold water, and spores were stored in water at 4°C at an optical density at 600 nm (OD_600_) of 10–30 (~1 to 3 × 10^9^ spores/mL) protected from light. Spores prepared on plates were scraped into cold water and then purified and stored as described above. All spores used in this work were >98% free from germinated spores or cells, with no debris seen in liquid or on surfaces of pelleted spores by phase contrast microscopy, in contrast to the case with less well-purified spores (13). Some spores were also prepared with 50 mM Laurdan in the liquid medium or 250 mM Laurdan in the plates to label spores’ IM, with the fluorescence properties of the incorporated dye used to assess IM fluidity (15, 16).

### Spore germination

Purified spores without Laurdan were germinated with the germinant receptor (GR) germinants 10 mM-valine, or a mixture of 10 mM each of L-asparagine, D-glucose, D-fructose, and KCl (AGFK). Prior to germination, spores at an OD_600_ of ~10 were heat activated at 70°C for either 30 min (L-valine germination) or 90 min (AGFK germination) (17). The activated spores were then cooled and used on the same day for germination. L-valine and AGFK germination were at 37°C with spores at an OD_600_ of ~0.5 in ~500 µL of 25 mM K-HEPES buffer (pH ~7.4) plus 50 µM TbCl_3_ and germinants. Germination was started by spore addition to the germination mix, and germination was measured by monitoring CaDPA release from germinating spores by terbium–dipicolinic acid (Tb-DPA) fluorescence in a fluorometric plate reader as described (18). Fluorescence at each time point was measured in duplicate, and values were averaged.

Spores were also germinated with 1 mM dodecylamine, a germinant that does not act on GRs (3, 19). Notably, dodecylamine germination is sensitive to spores’ IM fluidity, presumably because dodecylamine triggers germination by opening the IM SpoVAC protein channel that releases CaDPA (3, 20). Indeed, rates of dodecylamine germination increase higher in spores with a more fluid IM and decrease in spores with greater IM rigidity (20–23). This germination was at 40°C in ~4 mL of 25 mM Tris-HCl, pH 8, with spores at an OD_600_ of 0.5 and with 1 mM dodecylamine (19, 22). Germination was initiated by spore addition, and at various times, 450 µL was mixed with 50 µL of 500 µM TbCl_3_, and Tb-DPA fluorescence was measured. Again, duplicate values were taken at each time point, and duplicates were averaged.

### Spore resistance

Spore resistance to wet heat, UV radiation, formaldehyde (HCHO), hydrogen peroxide (H_2_O_2_), and sodium hypochlorite (NaOCl) was measured as described (21–26). Spore viability measurements at each time point were in duplicate, and all experiments were carried out twice on aliquots of stored spores; thus, all final data for these analyses were averages of four individual measurements.

Conditions for spore treatments were as follows, with spores incubated at an OD_600_ of ~1 (~10^8^ spores/mL) in 1 mL of treatment solution but 3 mL for UV treatment: (i) wet heat treatment was at 93°, and at various times, 50 µL aliquots were added to 450 µL of sterile cold water; (ii) HCHO: spores were incubated at 30°C in 2.5% HCHO in water, and 50 µL samples taken at various times were diluted in 450 µL of sterile 0.1 M glycine (pH 7.4) to inactivate the HCHO and dilutions incubated for 30 min at 23°C; (iii) NaOCl: spores were incubated at 23° in NaOCl with 2.5% available chlorine, and 50 µL aliquots were added to 450 µL of sterile 25 mM sodium thiosulfate to inactivate NaOCl and samples incubated for 30 min at 23°C; (iv) H_2_O_2_: spores were incubated at 23°C in 11% H_2_O_2_ in sterile 25 mM K-HEPES buffer at pH 7.4, and 50 µL samples were diluted in 450 µL of catalase (~5 µg/mL) in sterile 10 mM KHPO_4_ buffer, pH 7.5, and incubated for 30 min at 23°C to inactivate the H_2_O_2_; and (v) UV light: 3 mL spores at an OD_600_ of 1 in sterile water at 23°C were added to an ~2.5 cm diameter sterile plastic dish without a cover that was 27 cm below a UV lamp (UVP UVG-11, Analytik Jena USA) maximally emitting at 254 nm with ~100 mW/cm^2^, and 50 µL samples taken at various times were serially diluted 10-fold in sterile water to 10^−6^ as were all 1/10 dilutions obtained as described in conditions i–iv above. For all dilutions, 10 µL aliquots were spotted in duplicate in a grid on LB agar medium plates (23, 25); spots were allowed to dry; and plates were incubated at 30°C overnight and then at 37°C until no more colonies appeared and colonies were counted.

Graphs of spore viability versus incubation time were plotted using Excel. All data points were means of four individual measurements, and the error bars show the standard error of the mean. For statistical comparisons, two-tailed Student’s *t*-tests with unequal variance were performed, and *P* values of ≤0.05 were considered statistically significant. The viability of 30- and 60-day spores incubated in liquid was compared to the viability of 3-day spores incubated in liquid across all time points. Similarly, the viability of 30- and 60-day spores incubated on plates was compared to the viability of 3-day spores incubated on plates across all time points.

### Determination of relative spore IM fluidity and permeability

Relative spore IM fluidity was assessed by the fluorescence properties of the dye Laurdan incorporated into spores’ IM (15, 16, 23). These Laurdan-labeled spores were prepared by sporulating cells in 2×SG liquid or on plates containing either 50 mM Laurdan (15, 16) in liquid medium or 250 mM Laurdan in sporulation plates, the latter being necessary because preliminary work showed that the higher concentration was essential to get sufficient Laurdan uptake by spores made on plates. Sporulation was monitored by observing cells under a phase contrast microscope, and >90% sporulation was observed at 3 days. About one-third of the spores made in liquid medium and one-half of spores made on plates for 3 days were harvested, and the rest were incubated at 37°. After 30 days, the remaining spores made on plates were harvested, as were another third of the spores made in liquid, and the rest were incubated in liquid for 60 days and then harvested. All harvested spores were washed multiple times with water, decoated with urea in a few cases (27, 28) (see Results), and washed again. Samples were placed on agarose-coated glass microscope slides, and Laurdan fluorescence intensity was measured with a Zeiss LSM880 Confocal Laser Scanning Microscope following excitation at 405 nm and emission at 440 and 490 nm. Fiji software was used to obtain intensity values, using the mean function, of 30–80 individual spores. After background subtraction of unlabeled spores’ fluorescence, generalized polarization (GP), a measure of IM fluidity, was calculated using the formula GP = (I440 – I490) / (I440 + I490) (15, 16).

### Comparison of core CaDPA and water content and IM permeability in spores prepared in liquid or on plates for different times

To compare CaDPA content in spores prepared differently, equal numbers of spores, ~2 × 10^8^ as determined by counting in a Petroff-Hauser chamber, were boiled in 2 mL of water for 15 min, cooled briefly on ice, and centrifuged in a microcentrifuge, and the supernatant fluid was transferred to a new test tube, all as described (27–29). Two aliquots of 5, 10, and 25 µL of the supernatant fluid from each spore sample were separately added to sufficient 25 mM K-HEPES buffer (pH 7.4) with TbCl_3_ to give a total of 200 µL of 25 mM buffer and 50 µM TbCl_3_, and Tb-DPA fluorescence was measured in a fluorometric plate reader. All values were corrected to assays of 25 µL, and values for all samples were averaged. Finally, the values of each individual sample were compared to that for the average of all of them for a particular spore type.

Relative core wet densities, and thus core water content of different spore preparations, were determined by where intact spores banded upon centrifugation in a Nycodenz density gradient (Fig. S1) as described previously (30, 31). Spore IM permeability was measured by monitoring ^14^C-methylamine uptake by 3- and 30-day spores prepared in liquid or on plates as described (23).

## RESULTS

### Spore resistance

**Fig 1:**
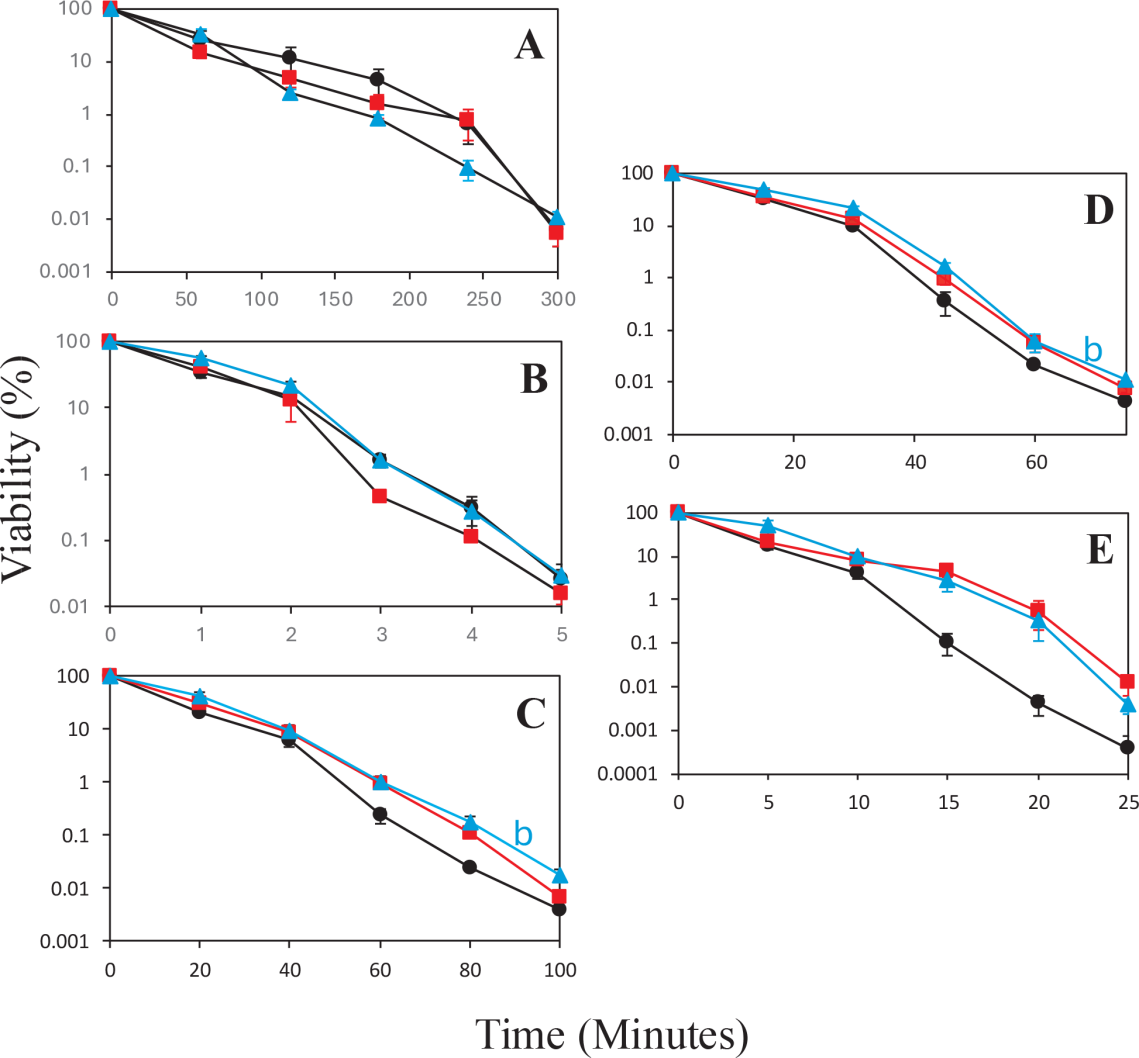
(**A–E**) Resistance of *Bacillus subtilis* spores made in liquid for different times to various agents. Spores prepared in liquid for various times were purified and exposed to (**A**) wet heat, (**B**) UV radiation, (**C**) HCHO, (**D**) H_2_O_2_, or (**E**) NaOCl, and spore survival was determined at various times, all as described in Materials and Methods. The data points at each time represent the log-transformed average viability of four measurements on each spore sample, with error bars indicating the standard error of the mean. (**A and B**) Statistically significant differences (*P* ≤ 0.05) between the 3-day sample and the (**A**) 30-day sample or (**B**) the 60-day sample. Statistically significant differences were detected for the 60-day sample relative to the 3-day samples in panels **C** and **D**. Symbols used for the spores prepared for different times were black lines and black filled circles for 3-day spores, red lines and solid red squares for 30-day spores, and blue lines and solid blue triangles for 60-day spores.

**Fig 2:**
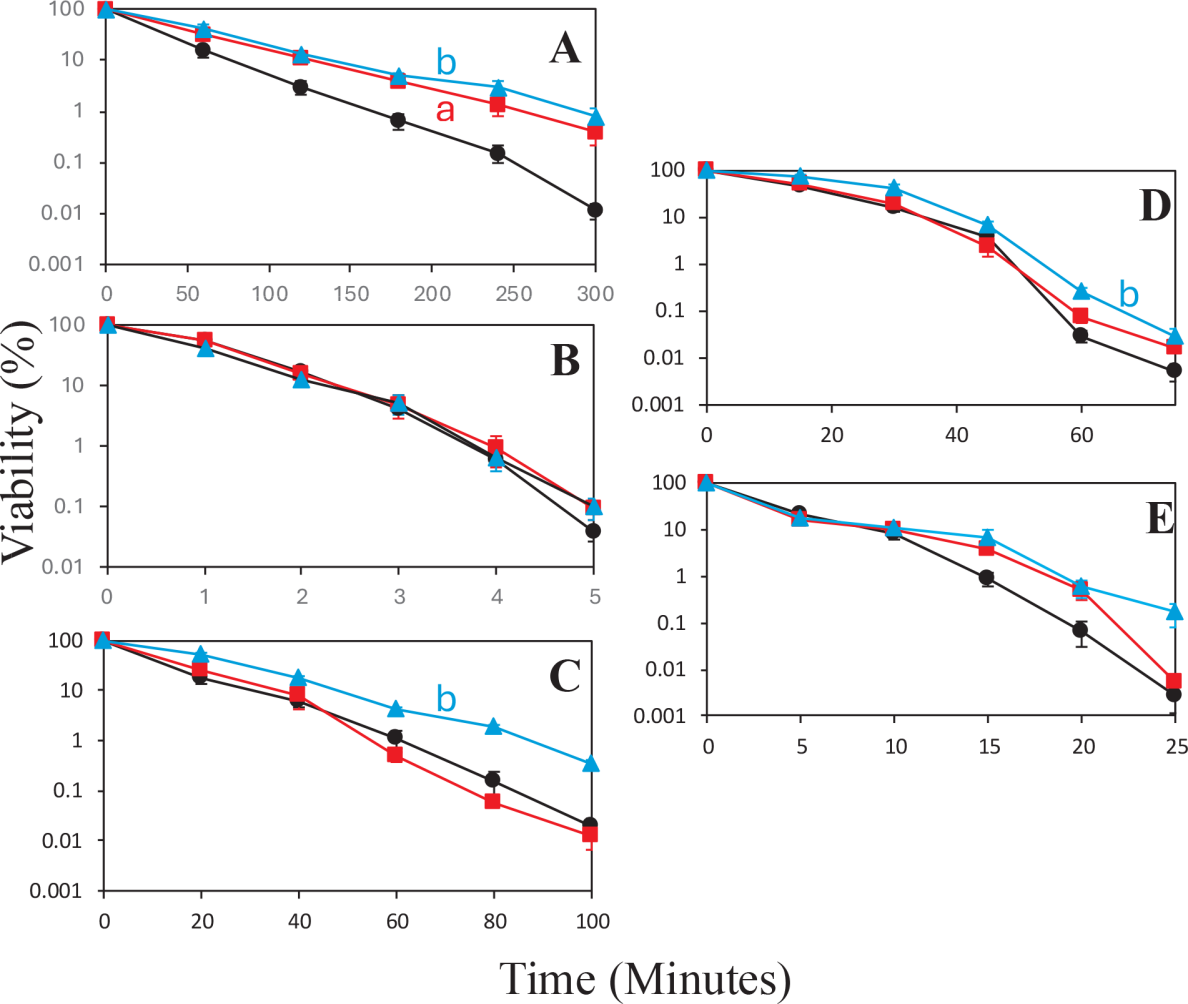
(**A–E**) Resistance of *B. subtilis* spores made on plates for various times to different agents. Spores prepared for 3 (●), 30 (〇), or 60 (▲) days on plates were exposed to (**A**) wet heat, (**B**) UV radiation, (**C**) HCHO, (**D**) H_2_O_2_, or (**E**) NaOCl, and spore survival was determined at various times, all as described in Materials and Methods. The data points at each time represent the log-transformed average viability of four measurements on each spore sample, with error bars indicating the standard error of the mean. (**A and B**) Statistically significant differences (*P* ≤ 0.05) between the 3-day sample and the (**A**) 30-day sample and (**B**) the 60-day sample. Statistically significant differences were detected for the 30- and 60-day samples in panel A, the 60-day sample in panel C, and the 60-day sample in panel D. Symbols used for the spores prepared for different times were black lines and black-filled circles for 3-day spores, red lines and solid red squares for 30-day spores, and blue lines and solid blue triangles for 60-day spores.

Spores prepared in liquid or on plates for 3, 30, or 60 days at 37°C were tested for their resistance to wet heat, UV radiation, HCHO, H_2_O_2_, and NaOCl (Fig. 1A through E, Fig. 2A through E) as described in Materials and Methods. There were no significant increases in resistance to wet heat, UV radiation, or NaOCl after 30- or 60-day incubation in liquid compared to after 3-day incubation in liquid (Fig. 1A, B, and E). However, there were slight but significant increases in HCHO and H_2_O_2_ resistance in 60-day spores prepared in liquid compared to 3-day spores prepared in liquid (Fig. 1C and D).

The results for spores prepared on plates were similar in some respects, as again there were no differences in UV radiation or NaOCl resistance with spores incubated for 30 and 60 days compared to 3 days (Fig. 2B and E). There were, however, significant increases in resistance to wet heat in the 30- and 60-day spores compared to the 3-day spores (Fig. 2A) and smaller but still significant increases in HCHO and H_2_O_2_ resistance in 60-day spores compared to that of 3-day spores (Fig. 2C and D).

### Spore germination

**Fig 3:**
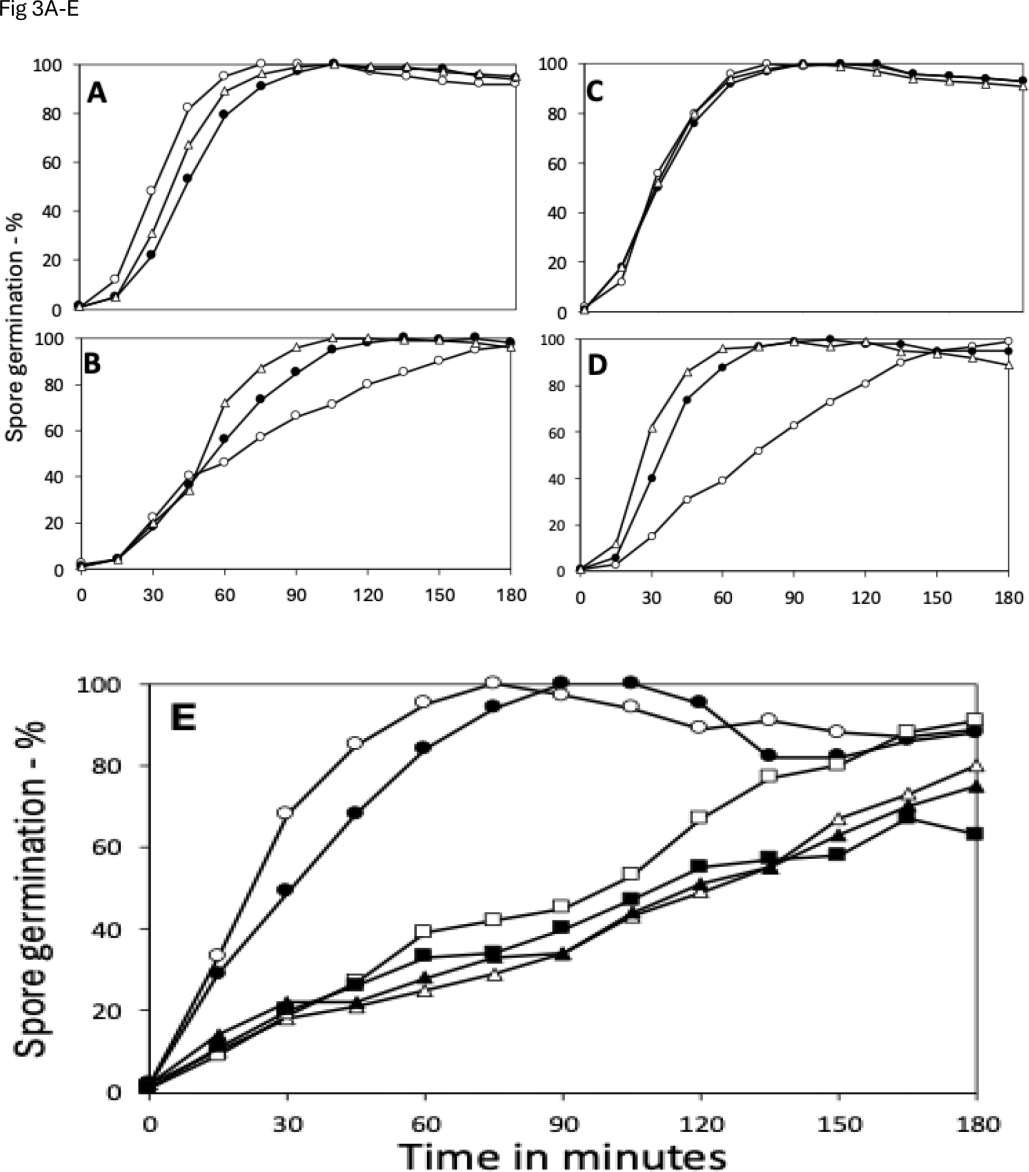
(**A–E**) Germination of spores made in liquid or plates and with different germinants. Spores prepared in liquid (**A and B**) or on plates (**C and D**) for 3 (〇), 30 (●), or 60 (△) days were heat activated and germinated with (**A and C**) L-valine, (**B and D**) AGFK, or (**E**) spores prepared in liquid (〇, △, and □) or on plates (●, ▲, and ■) for 3 days (〇 and ●), 30 days (□ and ■), or 60 days (△ and ▲) without heat activation were germinated with dodecylamine. In all cases, spore germination was measured as described in Materials and Methods. All values are averages of duplicate measurements at each time point (4 data points).

Increases in spore resistance to wet heat, HCHO, and H_2_O_2_ can be caused at least in part by changes in spores’ IM, in particular changes in IM fluidity or permeability (21–25). Since many proteins involved in spore germination are in or on the IM (3), changes in spores’ IM during long sporulation times might result in changes in spore germination. Consequently, the germination of spores prepared in liquid or on plates for different times was tested, and with both GR and GR-independent germinants (Fig. 3A through E). There were no notable differences in the L-valine germination rates of spores incubated in liquid or on plates for 3, 30, or 60 days via the GerA GR (Fig. 3A and C). However, with the AGFK mixture that requires cooperative interaction between the GerB and GerK GRs, the 30- and 60-day spores germinated faster than the 3-day spores, with this difference being the largest, with spores prepared on plates (Fig. 3B and D). However, the reason for these differences in AGFK germination is not clear.

**Fig 4:**
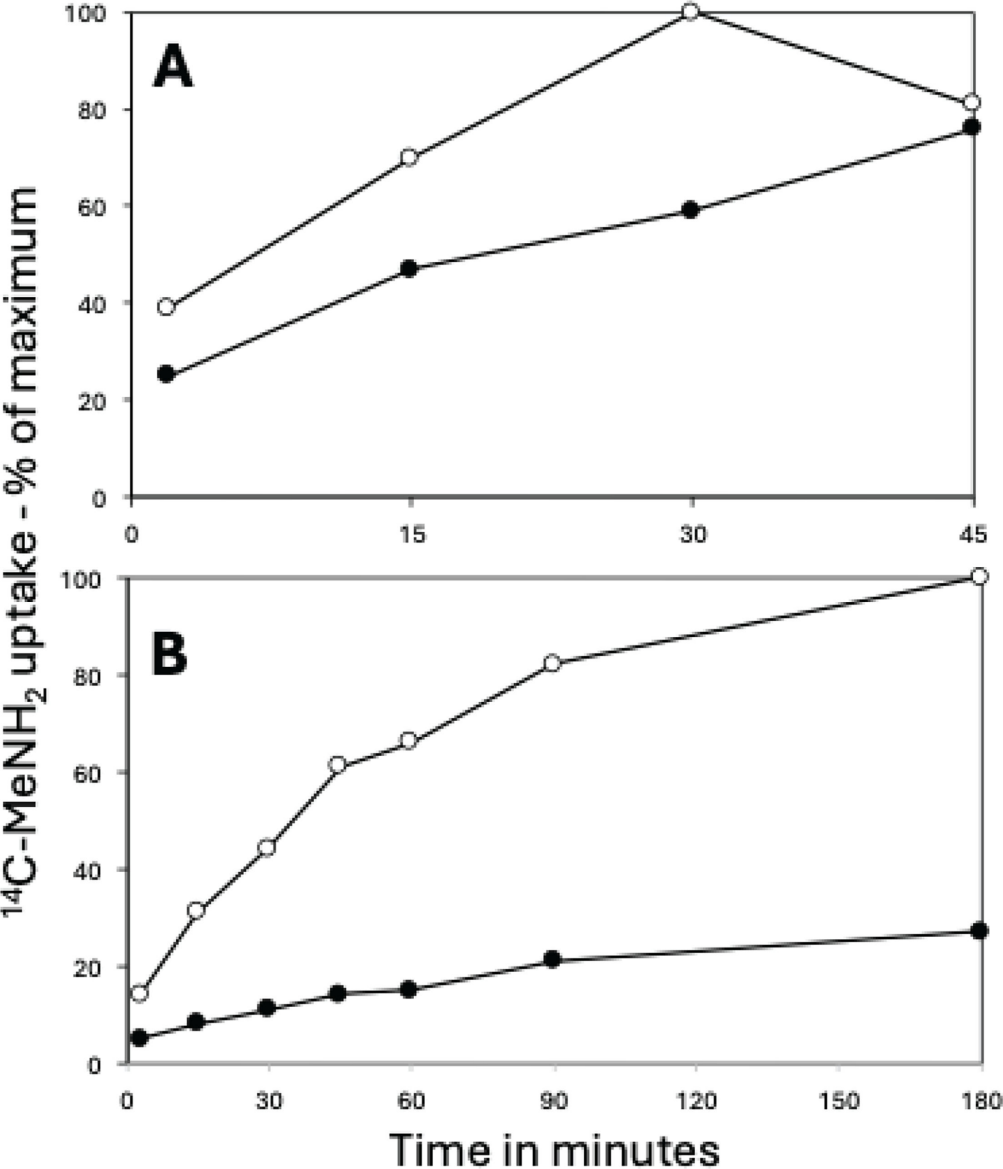
(**A and B**) IM permeability of 3- and 30-day PS832 spores prepared in liquid (**A**) or on plates (**B**). Spores’ IM permeability was measured by determining the percentage of spores’ uptake of ^14^C-methylamine as a function of time as described in Materials and Methods. All values shown are averages of results in two separate experiments. Symbols used are (**A**) 〇 and ●, spores prepared in liquid for 3 or 30 days, respectively, and (**B**) 〇 and ●, spores made on plates for 3 or 30 days, respectively.

Dodecylamine germination does not proceed via GRs, but rather directly opens the IM CaDPA channel (3). Notably, the 3-day spores prepared in liquid or on plates germinated more rapidly with dodecylamine than did spores incubated for 30 days, while the 60-day spores’ germination was slightly less rapid (Fig. 4). This is consistent with the 30- and 60-day spores having a more rigid IM than the 3-day spores. That increased IM rigidity decreases rates of dodecylamine germination has been seen previously using spores with and without specific IM proteins that increase IM rigidity (19–22).

### Other spore properties

As noted above, the fluidity of spores’ IM as well as the spore core’s content of water and CaDPA play roles in spore resistance and germination (21–24). Consequently, we examined these parameters in spores prepared for various times in liquid or on plates. In the case of CaDPA, it is clear that having minimal, if any, CaDPA in the spore’s core leads to higher core water contents, lower spore wet heat resistance, and altered germination, among other effects (26, 28). Thus, relative levels of CaDPA in spores made in liquid or on plates for 3, 30, or 60 days were determined (Table 1), and all six sets of spores analyzed had essentially the same CaDPA level. Thus, it seems unlikely that differences in CaDPA levels are important in affecting properties of the spores analyzed in the current work. A second spore core variable is water content, which has major effects on spore wet heat resistance and perhaps on other spore resistance properties (3). However, again, there were no significant differences in core water content in spores made for various times in liquid or on plates, as determined by density gradient centrifugation and using intact or decoated spores (Table 1; Fig. S1), also ruling out this parameter as having a role in the differences in resistance and germination properties between spores prepared longer and in the different conditions used in the current work.

**TABLE 1:** Relative CaDPA and core water levels in spores made in liquid or on plates for 3, 30, or 60 days^*a*^^*,**b*^

Sporulation in/on	Liquid medium			Solid medium		
Spores incubated for (day)	3	30	60	3	30	60
CaDPA level, average (%)^*c*^	102	104	97	101	97	98
Intact spores Nyc%^*d*^^*,**e*^	66	67	64	63	64	64

^*a*^ CaDPA analysis. Spores (2 × 10^8^) of two aliquots of purified spores, as determined by counting in a Petroff-Hauser chamber, were boiled for 15 min, cooled, and centrifuged, and duplicate 5, 10, and 25 µL aliquots of the supernatant fluids were assayed for CaDPA as described in Materials and Methods. All values were corrected for the different volumes assayed. Values for all six different spore samples were averaged, and values for individual samples are expressed as the percentage of the average value for the samples from all six types of spore samples analyzed. These values are all ≤±6%.

^*b*^ Core water analysis. Relative core water content in spores was determined by comparing banding positions in Nycodenz gradients as described in Methods and seen in Fig. S1.

^*c*^ Values are the levels of CaDPA in the spore core relative to the highest level that was set as 100%.

^*d*^ Values are the % Nycodenz (Nyc) banding positions of spores in Nycodenz density gradients (see Fig. S1).

^*e*^ Essentially identical results were obtained when decoated spores were used (data not shown).

An alternative cause of some of the differences in resistance and germination seen in spores prepared for different lengths of time and in different media is changes in spores’ IM. Indeed, increasing IM rigidity and decreasing permeability can significantly increase spore resistance to wet heat and to the chemicals HCHO and H_2_O_2_, and this can also decrease rates of dodecylamine germination (19, 20). Consequently, the IM permeability of ^14^C-methylamine into 3- and 30-day spores made in liquid or on plates was measured (Fig. 4A and B). Notably, spores made in liquid for 3 or 30 days had higher permeability to methylamine than did spores made on plates. To assess the relative IM rigidity in spores made for different times in liquid or on plates, we prepared spores with the dye Laurdan incorporated in the IM and then measured these spore fluorescence properties, in particular this dye’ GP, as a measure of IM fluidity/rigidity (15, 23, 32). Notably, the longer sporulating cells were incubated on plates, the more rigid the IM became, as shown by the increased GP value in 30-day compared to 3-day spores (Table 2) (32). In contrast, the GP values for spores prepared in liquid decreased significantly in 30-day compared to 3-day spores (Table 2). That the IM is less fluid in spores made on plates was also shown by the slower spore germination with dodecylamine of these spores. Higher IM rigidity in spores incubated longer on plates is also consistent with 30-day spores’ higher resistance to wet heat, perhaps because wet heat kills spores by inactivating IM enzymes involved in ATP generation when spores germinate (23). Importantly, in order for both H_2_O_2_ and HCHO to kill spores, these chemicals must cross the IM to damage targets in the spore core (2), so a more rigid IM presumably reduces the permeability of the IM to these chemicals. Note that hypochlorite does not kill spores by damaging core components (26). Remarkably, the 30-day spores made on plates had much lower IM permeability to methylamine than the 3-day spores (Fig. 4A and B), consistent with the 30-day spores’ higher HCHO/H_2_O_2_ resistance. There was a much smaller effect of sporulation time on methylamine permeability with spores made in liquid, although the 3-day spores prepared on plates had lower methylamine permeability than the 3-day spores prepared in liquid (compare Fig. 4A and B).

**TABLE 2:** Analysis of IM fluidity using Laurdan-labeled spores made in liquid or on plates^*a*^

Spores examined	Gp = (I440 − I490) / (I440 + I490)
Spores made in liquid	
PS832 (3 days)	0.22 ± 0.04 (35 spores)
PS832 (30 days)	0.08 ± 0.04 (35 spores)
PS832 (60 days)	0.06 ± 0.04 (30 spores)
Spores made on plates	
PS832 (3 days)	−0.18 ± 0.08 (80 spores)
PS832 (30 days)	+0.22 ± 0.05 (80 spores)

^*a*^ Spores labeled with Laurdan in liquid 2×SG medium and decoated or on plates were purified but not decoated, and their Laurdan fluorescence intensities from spores with excitation at 405 nm and emission at 440 and 490 nm were determined, allowing GP values to be calculated as described in Materials and Methods.

## DISCUSSION

There have been a number of publications comparing effects of *B. subtilis* sporulation on plates and in liquid on spore properties (6–9, 33). The properties examined in the previous work included spore resistance and GR-dependent germination. Notably, several studies found that *B. subtilis* spore wet heat resistance is increased significantly by either longer sporulation times (7) or on plates instead of in liquid (6), as in the current work. Analyses of core water and CaDPA content in these published studies were identical, even in spores with higher wet heat resistance, just as in the work reported in this paper. Thus, lower levels of core water or changes in CaDPA content are ruled out as causes of elevated wet heat resistance in spores incubated for long times on plates. Notably, *B. subtilis* spores lacking all coat material and prepared on plates also have significantly lower wet heat resistance than wild-type spores, but again the coatless spores had the same core water content as the wild-type spores (34). Thus, a major factor in spore wet heat resistance, spore core water content, plays no role in the altered wet heat resistance in a number of sporulation conditions and with spores ± coats.

An important factor in spore wet heat resistance that has not been as well studied as core water content is spore maturation after release from the sporangium, most likely by increased cross-linking of spore coat proteins (8, 9). While this causes increased wet heat resistance, in at least one case, there was no effect of this maturation and any coat-cross-linking on spore core water content (8). Notably, almost all studies noted above used a relatively similar rich medium for sporulation (6–8). One (9) used a more minimal medium, but even then, there was an ~5-fold increase in spore coat heat resistance going from 2- to 8-days spores prepared in a liquid medium.

A key question is, of course, what causes the large differences in spore wet heat resistance when spores are prepared for different times and in liquid or on plates. Factors involved in spore wet heat resistance include the core water and CaDPA content, the levels of DNA protective small, acid-soluble DNA binding proteins (SASP), and spore IM rigidity. In at least one case where spores were prepared in liquid or on plates, with the plate spores having significantly higher wet heat resistance, there was no detectable difference in DNA-binding SASP levels in the spores prepared in liquid or in plates (8). This leaves only IM rigidity as a possible explanation for the higher wet heat resistance of spores prepared on plates and/or for longer times. One measure of spore IM rigidity is spores’ germination with the non-GR determinant dodecylamine, which opens the IM CaDPA channel by interaction with the channel protein SpoVAC. Presumably with a more rigid IM, opening the SpoVAC CaDPA channel is more difficult than if the IM is more fluid. Indeed, dodecylamine germination was markedly slower in 30- and 60-day spores prepared in liquid or on plates compared to that in 3-day spores prepared similarly (Fig. 3E). In addition, spores made on plates have lower permeability to a variety of compounds, and IM permeability decreases more with longer sporulation times, albeit less so in spores prepared in liquid than on plates (Fig. 4).

A remaining question then is what is happening during longer sporulation and after spores’ release from the mother cell, more so on plates than in liquid, to decrease IM fluidity and thus causing larger effects on spore properties. Currently, the only major event known to take place following spores’ release from mother cells is increased spore coat cross-linking, which can certainly affect spore wet heat resistance (8–10, 12). However, what specific cross-linking is involved and why it may be increased are not clear and need further detailed study.

### Conclusions

This work has confirmed that longer sporulation time on plates, but less so in liquid, gives spores with higher resistance to wet heat and to chemicals that must cross spores’ IM to cause spore killing. Importantly, the IM in spores prepared on plates, but less so in liquid, is (i) more rigid and (ii) less permeable than in spores prepared in liquid even for long times or prepared on plates for short times, and this is most likely the reason for the higher resistance of spores prepared on plates. However, levels of core water and CaDPA in spores prepared in liquid or on plates were essentially identical, indicating that these core parameters play no role in generating a more rigid and impermeable IM. Unfortunately, precisely how longer sporulation on plates promotes the latter spore properties is not yet clear.

## Data Availability

The data supporting the results in this article will be shared on reasonable request to the corresponding author.

## SUPPLEMENTAL MATERIAL

Figure S1 jb.00389-25-s0001.tif Nycodenz density gradients.

ASM does not own the copyrights to Supplemental Material that may be linked to, or accessed through, an article. The authors have granted ASM a non-exclusive, world-wide license to publish the Supplemental Material files. Please contact the corresponding author directly for reuse.
