# Supplementary figures and images for: Effects of sporulation times in liquid or on plates on Bacillus subtilis spore resistance, germination, inner membrane fluidity and permeability, and core contents
Source: J Bacteriol. 2025 Nov 14;207(12):e00389-25. doi: 10.1128/jb.00389-25 (PMC12713371; doi:10.1128/jb.00389-25)

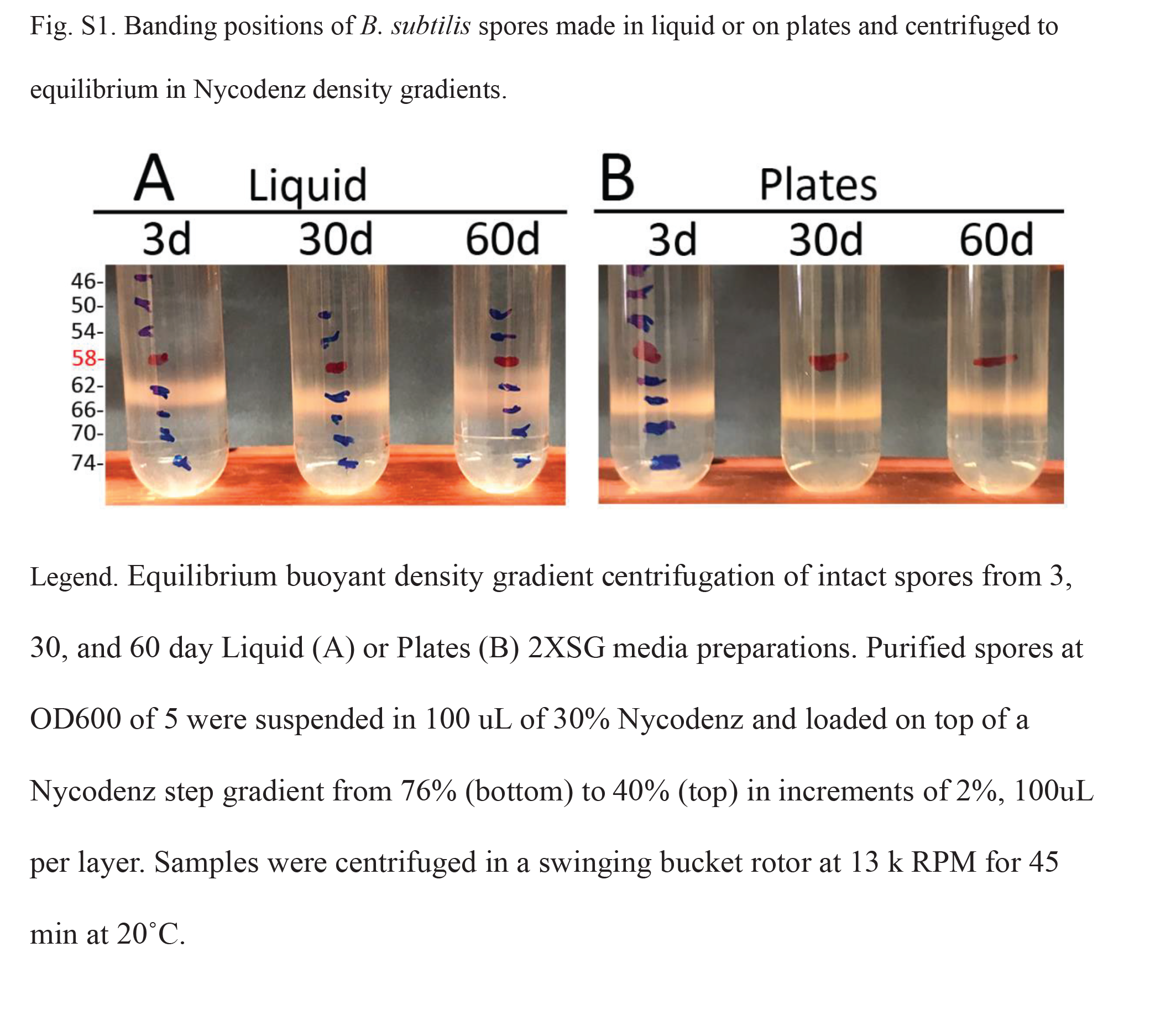

Supplement: Figure S1 — Nycodenz density gradients. [file jb.00389-25-s0001.tif]
